# Identification of Hub Genes and Immune Infiltration in Psoriasis by Bioinformatics Method

**DOI:** 10.3389/fgene.2021.606065

**Published:** 2021-02-03

**Authors:** Wenxing Su, Yuqian Wei, Biao Huang, Jiang Ji

**Affiliations:** ^1^Department of Dermatology, The Second Affiliated Hospital of Soochow University, Suzhou, China; ^2^Department of Medicine, Soochow University, Suzhou, China; ^3^Department of Burn and Plastic Surgery, The First Affiliated Hospital of Soochow University, Suzhou, China

**Keywords:** psoriasis, bioinformatics analysis, differentially expressed genes, hub genes, immune infiltration

## Abstract

**Background:**

Psoriasis is a chronic, prolonged, and recurrent skin inflammatory disease. However, the pathogenesis of psoriasis is not completely clear, thus we aimed to explore potential molecular basis of it.

**Methods:**

Two datasets were downloaded from the Gene Expression Omnibus database. After identifying the differentially expressed genes of psoriasis skin lesion samples and healthy controls, three kinds of analyses, namely functional annotation, protein-protein interaction (PPI) network, and immune infiltration analyses, were performed.

**Results:**

A total of 152 up-regulated genes and 38 down-regulated genes were selected for subsequent analyses. Evaluation of the PPI network identified the most important module containing 13 hub genes. Gene ontology analysis showed that the hub genes have a significant enrichment effect on positive regulation of cell migration, defense response to the other organism and epithelial cell differentiation. KEGG signaling pathway analysis showed that the hub genes were significantly enriched in chemokine signaling, Toll-like receptor signaling pathway, and IL-17 signaling pathway. Compared with the normal control sample, naive B cells, CD8^+^ T cells, activated memory CD4^+^ T cells, follicular helper T cells, gamma delta T cells, resting NK cells, monocytes, M0 macrophages, M1 macrophages, activated dendritic cells and neutrophils infiltrated more, while memory B cells, naive CD4^+^ T cells, regulatory T cells (Tregs), activated NK cells, resting mast cells, and eosinophils infiltrated less.

**Conclusion:**

To conclude, the hub genes and pathways identified from psoriasis lesions and normal controls along with the immune infiltration profile may provide new insights into the study of psoriasis.

## Introduction

Psoriasis is a chronic, prolonged, and recurrent skin inflammatory disease. The World Health Organization (WHO) reported in 2017 that the estimates of the prevalence of psoriasis in adults ranged from 0.51 to 11.43%, and children from 0 to 1.37% (Michalek et al., 2017). At present, there are greater than 6 million patients with psoriasis in China, seriously endangering the physical and mental health of the people. It is currently believed that its pathogenesis is related to numerous factors such as genetic susceptibility sites, infection, immune disorders, metabolic disorders, endocrine disorders, and environment (Armstrong and Read, 2020). However, the pathogenesis of psoriasis is not entirely clear. Therefore, to effectively control the clinical symptoms of psoriasis. It is in urgent need of revealing the molecular mechanism of its occurrence and development.

Bioinformatics analysis of gene expression profiles or other high-throughput data has played a critical role in studying the pathogenesis of human diseases in recent years. Adopting gene chips can quickly detect the expression information of all the genes within the same sample time-point, which is ideal for screening differentially expressed genes (DEGs; Wang, 2000). In the study, we downloaded the mRNA microarray dataset from the Gene Expression Omnibus (GEO), including lesions of 58 psoriasis patients, and normal epidermis of 64 healthy individuals. The DEGs of the dataset were screened and biological functions analysis along with pathway enrichment analysis was performed. Through protein-protein interaction (PPI) network analysis, we identified 13 hub genes. In addition, immune infiltration of lesions of psoriasis patients was analyzed by the method of CIBERSORT algorithm, which is widely used to assess the relative content of 22 kinds of immune cells. The purpose of this study was to identify key biomarkers of the DEGs and immune infiltration in lesions of psoriasis patients and to provide the corresponding therapeutic targets.

## Materials and Methods

### Data Collection

Gene Expression Omnibus^1^ is a gene expression database created by NCBI, which contains high-throughput gene expression data submitted by research institutes worldwide (Barrett et al., 2013). Two microarray datasets [GSE13355 (Nair et al., 2009) and GSE14905 (Yao et al., 2008)] were downloaded from GPL570 (HG-U133_Plus_2) Affymetrix Human Genome U133 Plus 2.0 Array. The annotation file within the platform is to match the probes to the corresponding genes. GSE13355 contains the mRNA information of lesions (LS) from 58 psoriasis patients and 64 normal skin (NS) from healthy individuals. GSE14905 contains 33 psoriasis LS, 28 non-lesion (NL), and 21 NS.

### Identification of DEGs

The raw data from GSE13355 dataset was read through the affy package of R Software, with the RMA algorithm serving for background correction and data normalization, and DEGs were screened out by the limma package. Probe sets without corresponding gene symbols or genes with more than one probe set were, respectively, removed or averaged. We performed a Benjamini–Hochberg test and *T*-tests to compute the false discovery rate (FDR) and *P*-value to identify the DEGs between psoriasis patients and healthy controls. | LogFC (fold change)| ≥1.5 and adjusted *P*-value < 0.01 were considered statistically significant.

### Enrichment Analyses

Gene set enrichment analysis (GSEA) refers to sorting genes according to the degree of differential expression from the two types of samples and then checking whether the preset gene set is enriched at the top or bottom of this sorting table (Subramanian et al., 2005). GSEA can retain this key information without screening out differences, and then find out those functional gene sets that are not obviously different but sharing the same trend of genetic differences. The genetic information of all psoriasis and healthy control samples was uploaded to GSEA for further analysis. The pathway enrichment analyses of DEGs were performed by KOBAS 3.0^2^ (Wu et al., 2006). Another two databases, KEGG pathway and Reactome, were served for further analyses. Conducting pathway analysis was to find out which cellular pathways may be involved in the changes of DEGs, thus, to further identify the key pathways related to DEGs. *P*-value < 0.05 was considered statistically significant. Funrich, a functional enrichment analysis tool was used here to analyze the biological pathways of DEGs (Pathan et al., 2015).

### PPI Network Construction and Hub Genes Selection and Analyses

An online database of known and predicted protein interactions, Search Tool for the Retrieval of Interacting Genes (STRING; http://string-db.org; version 10.0), was applied to predict the PPI network of DEGs (Franceschini et al., 2013) while interactions with a combined score > 0.4 were taken as statistically significant. Cytoscape (version 3.6.1, http://www.cytoscape.org; Smoot et al., 2011) was used to visualize molecular interaction networks, specifically using its plug-in CytoNCA to analyze the topological properties of nodes in the PPI network with parameters set as unweighted. By ranking the scores of each node, we obtained important nodes of protein interactions within the network. Considering most networks were scale-free, the hub genes with degree ≥ 20 were selected. Metascape^3^ was used to further verify the function enrichment of hub genes (Zhou et al., 2019) with *P*-value < 0.05 as the cutoff. Hub genes pathway analysis was performed and visualized by ClueGO (version 2.5.4) and CluePedia (version 1.5.4), also plug-ins of Cytoscape. *P*-value < 0.01 was considered to be statistically significant. A network of genes and their co-expression genes was analyzed via GeneMANIA^4^ (Warde-Farley et al., 2010). Finally, the expression of identified hub gene was verified in GSE14905 dataset.

### Evaluation of Immune Cell Infiltration

Since the CIBERSORT algorithm was used to analyze the normalized gene expression data obtained, the proportions of 22 kinds of immune cells were obtained. Then, we used ggplot2 package for PCA clustering analysis on immune cell infiltration matrix data, so to obtain a two-dimensional PCA clustering map. Corrplot package was used to draw a correlation heatmap to visualize the correlation of 22 types of infiltrating immune cells while ggplot2 package to draw violin diagrams to visualize the differences in immune cell.

### Correlation Analysis of Hub Genes and Infiltrating Immune Cells

Spearman correlation analysis was performed on hub genes and infiltrating immune cells by ggstatsplot package with the results being visualized via ggplot2 package.

### LCN2/IL17A Expression Level and Correlation Analysis With Hub Genes

LCN2 is a gene significantly expressed in psoriasis. On the one hand, as an antimicrobial peptide, it can fight bacterial infections by sequestrating iron (Flo et al., 2004); on the other hand, it can act as a trigger for neutrophil activation to enhance inflammation (Shao et al., 2016). As a pro-inflammatory factor, IL-17A can induce the expression of chemokines and inflammatory cytokines, and recruit neutrophils and monocytes to accumulate to cause inflammation. In recent years, IL-17A/IL-17RA monoclonal antibody drugs (such as secukinumab, ixekizumab, and brodalumab) have significant effects on psoriasis and also illustrate the core position of IL-17A in psoriasis (Frieder et al., 2018). In order to better explain the role of hub genes, we analyzed their correlation.

## Results

### Identification of DEGs in Psoriasis

**FIGURE 1:**
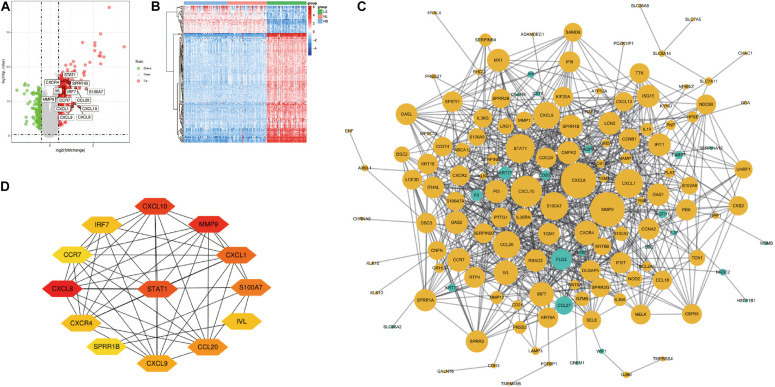
Volcano figure, heat maps, PPI network of DEGs. **(A)** Volcano figure of DEGs. Among them, red indicates up-regulated genes, and green indicates down-regulated genes. Gray indicates no differential expression. **(B)** The heat maps of DEGs. Red indicates up-regulated genes and blue indicates down-regulated genes. **(C)** The PPI network of DEGs was constructed using Cytoscape. Up-regulated genes are marked in light yellow; down-regulated genes are marked in light green. **(D)** The most important module composed of hub genes.

After the raw data is processed by R software, a total of 190 DEGs, consisting of 152 up-regulated genes and 38 down-regulated genes was identified between LS from psoriasis patients and NS from healthy individuals (Figure 1A). The heat map shows that these DEGs can clearly distinguish skin lesions from NLs and healthy tissues (Figure 1B).

### Analysis of the Functional Characteristics

**FIGURE 2:**
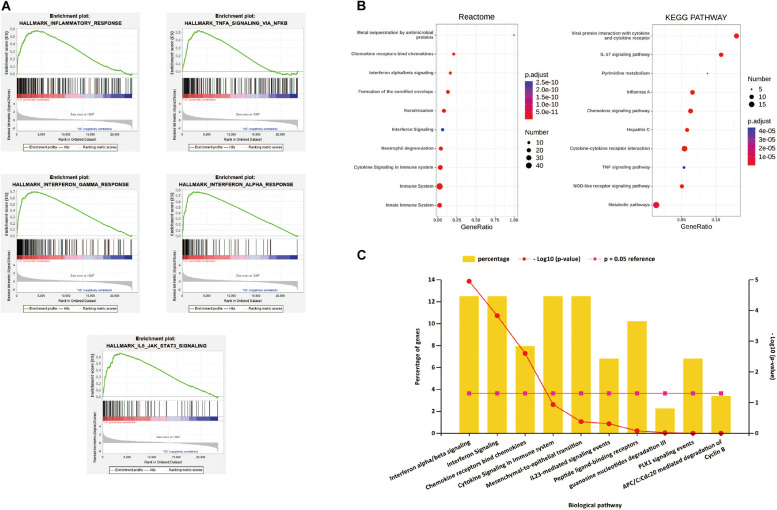
**(A)** h.all.v 6.2.symbols.gmt [Hallmarks] gene set database was used to analyze the whole gene expression value of the psoriasis and healthy controls samples. GSEA first filtered the gene set according to the number of genes contained in the gene set, with the minimum number of 15 genes, and the maximum number of 500 genes by default. Significant gene sets were cut-off by FDR < 0.25 and *P*-value < 0.05. **(B)** The pathway analysis of DEGs by KOBAS 3.0. The top 10 terms of the pathway enrichment result, from two databases “KEGG pathway” and “Reactome.” **(C)** The biological pathways of DEGs by Funrich. *P*-value < 0.05 was considered significant.

After all the gene expression information of GSE13355 is uploaded to the GSEA software, the hallmark gene set database is selected to analyze the enrichment entries on the overall gene expression level. The result showed that five gene sets were significantly enriched in psoriasis samples (Figure 2A), including inflammatory response, TNF-α signaling via NF-κB, IFN-α/β response and IL-6/JAK/STAT3 signaling.

The first 10 terms of the pathway enrichment results of the “KEGG pathway” and “Reaction” databases are shown in Figure 2B and Supplementary Tables 1,2. According to the enrichment analysis results of biological pathways performed by Funrich software, these 190 DEGs mainly enrich IFN-α/λ signaling and chemokine receptors bind chemokines, as shown in Figure 2C. These results indicate that immune response and inflammatory response play an indispensable role in the pathogenesis of psoriasis.

### PPI Network Construction and Hub Genes Selection and Analyses

**FIGURE 3:**
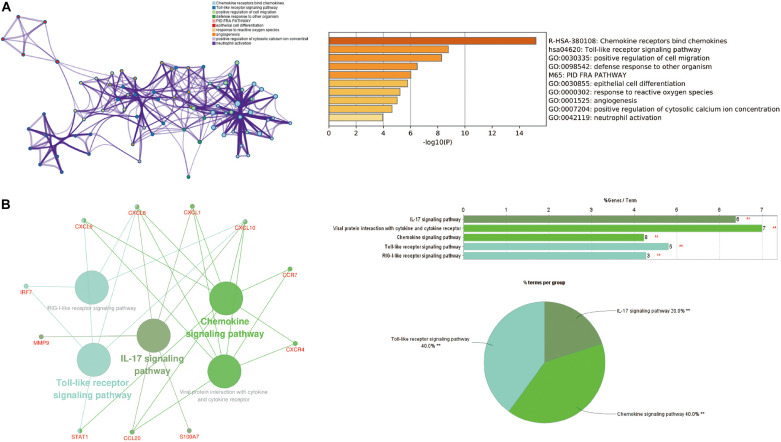
The function analysis of hub genes (*P*-value < 0.05). **(A)** The functions of hub genes were mainly enriched in positive regulation of cell migration, defense response to other organism, and epithelial cell differentiation. **(B)** The most significant pathway and related genes. The results show that these hub genes are mainly involved in chemokine signaling, Toll-like receptor signaling pathway, and IL-17 signaling pathway. **p < 0.01.

**FIGURE 4:**
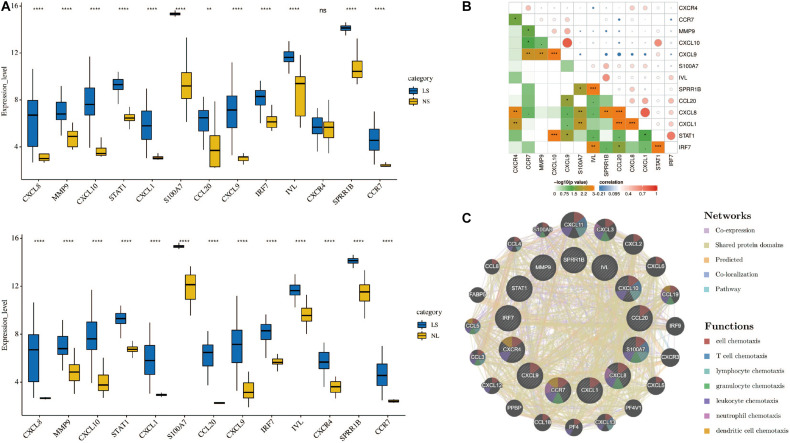
**(A)** Hub genes expression level in the dataset GSE14905. The comparison between the two sets of data uses the Student’s *t* test. Lesions (LS), normal skin (NS), and non-lesion (NL). **(B)** Correlation heat map of 13 hub genes. The circle in the upper right represents the correlation coefficient, red represents the positive correlation, and blue represents the negative correlation. The lower left part represents the *P* value. **(C)** Hub genes and their co-expression genes were analyzed using GeneMANIA. *p < 0.05; **p < 0.01; ***p < 0.001; ****p < 0.0001.

Cytoscape visualized the PPI network of DEGs, which contains 152 nodes and 762 interaction pairs (Figure 1C). A total of 13 genes (degree ≥ 20) was identified as hub genes (Figure 1D). Table 1 shows the detailed information of the key genes. Metascape functional annotation results show that hub genes were mainly enriched in chemokine receptors bind chemokines, Toll-like receptor signaling pathway and positive regulation of cell migration (Figure 3A). Similarly, ClueGO revealed that the most involved pathways were chemokine signaling, Toll-like receptor signaling pathway, and IL-17 signaling pathway (Figure 3B). In addition, the result of independence testing analysis suggested that all hub genes were significantly increased in psoriasis lesions, CXCR4 excluded (Figure 4A). Correlation analysis showed that CXCL9 and CXCL10 had the highest positive correlation with a Spearman’s correlation coefficient of 0.83 (Figure 4B). CXCL1 was also positively correlated with CXCL8 (*r* = 0.81, *p* = 1.78e–14). Finally, the analysis results from the GeneMANIA database show that 13 hub genes and their co-expressed genes constitute a complex PPI network with Co-expression of 63.02%, Shared protein domains of 19.61%, Predicted of 10.65%, Co-localization of 4.45%, and Pathway of 2.27% (Figure 4C).

**TABLE 1:** Details of 13 hub genes.

Gene symbols	Degrees	Full names	Gene function
CXCL8	41	C-X-C motif chemokine ligand 8	It is mainly secreted by neutrophils and acts as a chemokine to guide neutrophils to the site of infection
MMP9	40	Matrix metallopeptidase 9	Matrix metalloproteinase (MMP) family of proteins are mainly involved in the breakdown of extracellular matrix
CXCL10	34	C-X-C motif chemokine ligand 10	The binding of this protein to CXCR3 can include stimulating the migration of monocytes, natural killer cells and T cells, and regulating the expression of adhesion molecules
STAT1	30	Signal transducer and activator of transcription 1	Acts as a transcription factor to mediate the expression of multiple genes
CXCL1	29	C-X-C motif chemokine ligand 13	As a chemokine of neutrophils, it plays an important role in inflammation
S100A7	28	S100 calcium binding protein A7	The protein is overexpressed in hyperproliferative skin diseases, exhibits antimicrobial activities against bacteria and induces immunomodulatory activities
CCL20	27	C-C motif chemokine ligand 20	The protein shows chemotactic activity on lymphocytes and can inhibit proliferation of myeloid progenitors
CXCL9	26	C-X-C motif chemokine ligand 9	Combined with CXCR3, it can chemoattract lymphocytes instead of neutrophils
IRF7	22	Interferon regulatory factor 7	IRF7 has been shown to play a role in the transcriptional activation of virus-inducible cellular genes, including interferon beta chain genes
IVL	22	Involucrin	Involucrin, a component of the keratinocyte crosslinked envelope, is found in the cytoplasm and crosslinked to membrane proteins by transglutaminase
CXCR4	22	C-X-C motif chemokine receptor 4	It is a specific receptor for chemokine stromal cell-derived factor 1 (CXC12)
SPRR1B	20	Small proline rich protein 1B	The protein can be cross-linked with membrane proteins by transglutaminase to form an insoluble layer under the plasma membrane
CCR7	20	C-C motif chemokine receptor 7	This receptor-mediated signal can regulate T cell homeostasis in lymph nodes, and may also play a role in the activation and polarization of T cells and the pathogenesis of chronic inflammation

### Immune Cell Infiltration Results

**FIGURE 5:**
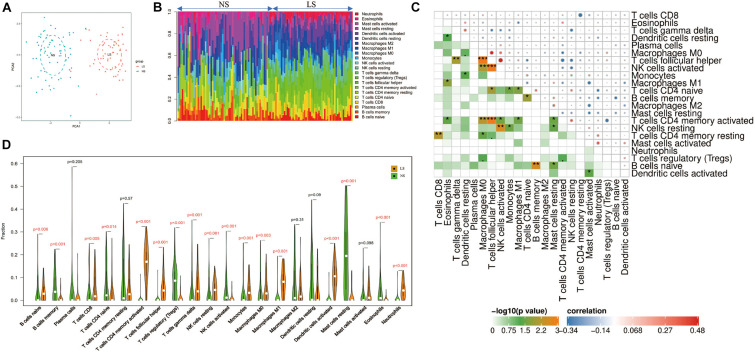
Immune cell infiltration mode. **(A)** PCA cluster graph of immune cell infiltration between the psoriasis sample and the control sample. **(B)** The relative percentage of 22 immune cell subpopulations out of 122 samples from the GSE13355 data set. **(C)** Related heat maps of 22 immune cells. The circle on the upper right represents the correlation coefficient, red represents positive correlation, and blue represents negative correlation. The lower left part represents the *P* value. **(D)** Violin chart of the ratio of 22 immune cells. The red mark represents a significant difference between the two groups of samples. *p < 0.05; **p < 0.01; ***p < 0.001.

Through the CIBERSORT algorithm, we analyzed the differences in immune infiltration of 22 immune cell subgroups in psoriasis LS samples and NS samples. The PCA cluster analysis of immune cell infiltration showed that there were significant differences in immune cell infiltration between LS samples and NS samples (Figure 5A). Figure 5B summarizes the results obtained from 58 psoriasis patients and 64 normal controls. Correlation heatmap of the 22 types of immune cells revealed that T cells follicular helper, activated NK cells, and M0 macrophages had a significant positive correlation. Activated CD4 memory T cells had a significant negative correlation with T cells follicular helper (Figure 5C). The violin chart shows that compared with the normal control sample, there are more naive B cells, CD8^+^ T cells, activated memory CD4^+^ T cells, follicular helper T cells, gamma delta T cells, resting NK cells, monocytes, M0 macrophages, M1 macrophages, activated dendritic cells and neutrophils in the psoriasis lesion tissue, but less memory B cells, naive CD4^+^ T cells, regulatory T cells (Tregs), activated NK cells, and resting mast cells and eosinophils (Figure 5D).

### Correlation Analysis of Hub Genes and Infiltrating Immune Cells

**FIGURE 6:**
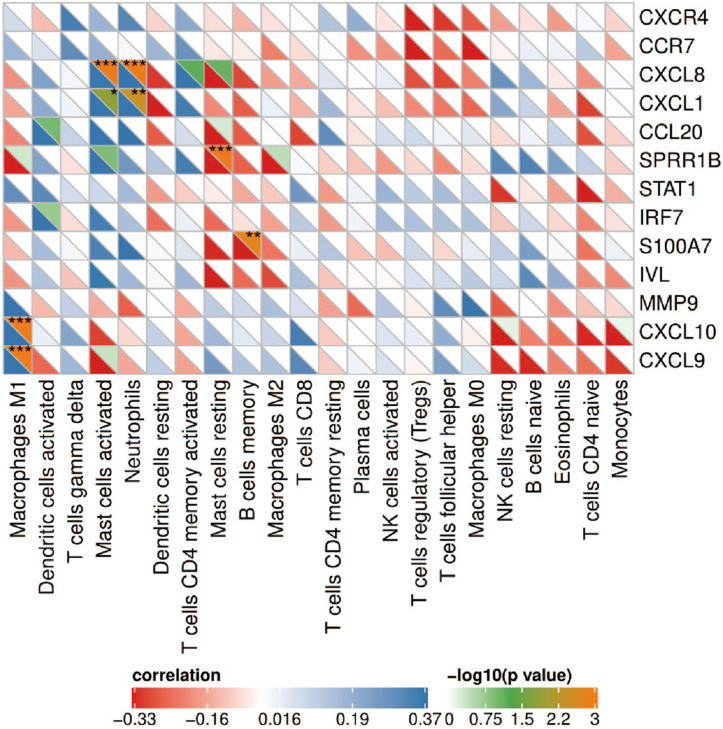
Correlation heat map between hub genes and immune cells. The upper right of the grid represents the *P* value, and the lower left represents the correlation coefficient. *p < 0.05; **p < 0.01; ***p < 0.001.

Correlation analysis showed that CXCL8 and CXCL1 were positively correlated with neutrophils (*r* = 0.579, *p* = 5.49e-4 and *r* = 0.539, *p* = 3.58e-3); CXCL9 and CXCL10 were positively correlated with M1 macrophages (*r* = 0.745, *p* = 5.72e-09 and *r* = 0.620, *p* = 5.82e-05); SPRR1B was negatively correlated with resting mast cells (*r* = –0.635, *p* = 2.51e-05); S100A7 was negatively correlated with memory B cells (*r* = –0.556, *p* = 1.64e-3; Figure 6). The correlation results showed that CXCL8 and CXCL1 jointly promoted the infiltration of neutrophils; CXCL9 and CXCL10 promoted the infiltration of M1 macrophages in the psoriatic lesions.

### LCN2/IL17A Expression Level and Correlation Analysis With Hub Genes

**FIGURE 7:**
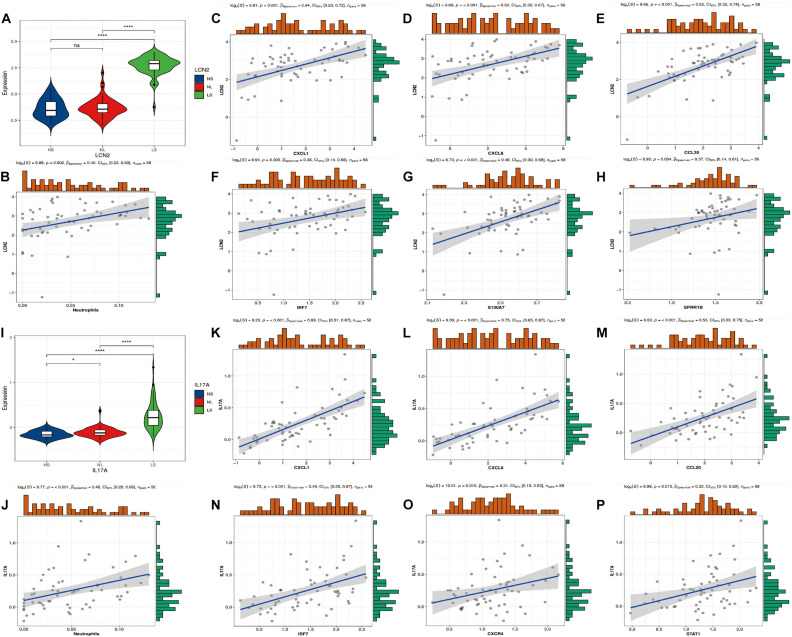
LCN2/IL17A expression level and correlation analysis with hub genes. **(A)** Expression level of LCN2. **(B)** The correlation between LCN2 and neutrophils. **(C–H)** The correlation between LCN2 and hub genes. **(I)** Expression level of IL17A. **(J)** The correlation between IL17A and neutrophils. **(K–P)** The correlation between IL17A and hub genes.

Consistent with previous studies, LCN2/IL17A is significantly highly expressed in psoriatic lesions (Figures 7A,I). In addition, we found that their expression was significantly positively correlated with the level of neutrophils (Figures 7B,J). Furthermore, four hub genes (CXCL1, CXCL8, CCL20, and IRF7) are positively correlated with LCN2/IL17A at the same time (Figures 7C–H,K–P).

## Discussion

As a chronic inflammatory skin disease that easily recurs, psoriasis has affected nearly 100 million people worldwide (Michalek et al., 2017). The resulting itching and pain, especially the abnormal appearance, make these patients prone to anxiety and depression, and even cause suicide (Egeberg et al., 2019). Although there are currently some treatments to control their symptoms, they cannot fundamentally solve the problem of high recurrence rates. Thus, exploring the molecular mechanism of its occurrence and development is in urgent need. Gene chip technology can reveal tens of thousands of genetic changes during disease development, which may provide promising therapeutic targets for diseases. In this study, we identified significantly DEGs by comparing gene expression profiles of psoriatic lesions with NS. Thirteen hub genes were screened by constructing a protein interaction network between them, and their biological functions and signal transduction pathways were analyzed. These hub genes were found to get involved in a variety of immune responses and immune cell chemotaxis, so we analyzed the immune cell infiltration in the lesions of patients with psoriasis using the CIBERSORT algorithm. The results showed that there was a significant difference between the immune cell infiltration in the lesions of psoriasis patients and that of the normal controls.

The results of gene enrichment analysis, which was performed using GSEA, KEGG, Reactome, and Funrich databases, showed that these genes were mainly involved in the inflammatory response, TNF-α signaling via NF-κB, interferon signaling pathway, IL-6/JAK/STAT3 signaling and chemokine receptors bind chemokines, revealing that immune response and inflammatory response play critical roles in pathogenesis of psoriasis. When TNF-α binds to its receptor, it can initiate the NF-κB signaling pathway to regulate the transcription of various cytokines, chemokines, adhesion molecules and enzymes, and aggravate the inflammatory response of psoriasis. In a number of guidelines, TNF-α inhibitors are considered to be the drugs of choice for plaque psoriasis and psoriatic arthritis (Armstrong and Read, 2020). However, TNF-α inhibitors may also cause serious adverse reactions, such as inactivation of tuberculosis during incubation, and increased risk of certain malignant tumors. A3AR, a G protein-coupled receptor, when activated, can downregulate the NF-κB signaling pathway and promote the apoptosis of inflammatory cells. It is expected to become the target of future treatment (Jacobson et al., 2018). After IFN-α/β and IL6 act on the corresponding receptors on keratinocytes, the JAK/STAT3 signaling pathway can be activated to induce the proliferation and differentiation of keratinocytes and activate cytokines. In randomized clinical trials, local JAK inhibitors have shown good efficacy in psoriasis patients and are expected to become a new and effecitve drug in treating psoriasis in the future (Hosking et al., 2018).

Grouping and organizing all the genes encoding proteins in the genome by constructing a PPI network has proved useful in the analysis of many diseases. Using CytoNCA in Cytoscape 3.6.1 to analyze the PPI results, 13 hub genes were obtained, including chemokines and chemokine receptors (CXCL8, CXCL1, CXCL9, CXCL10, CCL20, CXCR4, and CCR7), cytokines (MMP9, STAT1, S100A7, and IRF7), and envelope proteins (IVL, SPRR1B). Although there have been similar studies before (Zhang et al., 2019), they analyzed the lesions and NL of patients with psoriasis. Taking into account the influence of the autoimmune status of the same patient on the results, we compared the skin lesions of patients with psoriasis and matched healthy control skins. In addition, they did not further explore the immune cell infiltration state of the patient’s skin lesions. Based on the Metascape database, we found that the hub gene is mainly involved in biological processes such as positive regulation of cell migration, defense response to other organisms and epithelial cell differentiation. These results indicate that these hub genes play an important role in immune cell chemotaxis and epithelial cell differentiation in psoriasis. In order to further explore the role of immune cell infiltration in psoriasis, we used CIBERSORT to comprehensively evaluate the immune invasion of skin lesions.

The results show that, compared with the normal control sample, naive B cells, CD8^+^ T cells, activated memory CD4^+^ T cells, follicular helper T cells, gamma delta T cells, resting NK cells, monocytes, M0 macrophages, M1 macrophages, activated dendritic cells and neutrophils infiltrated more, while memory B cells, naive CD4^+^ T cells, regulatory T cells (Tregs), activated NK cells, resting mast cells, and eosinophils infiltrated less. The infiltration of these immune cells is consistent with previous reports (Clark and Kupper, 2006; Armstrong and Read, 2020). In addition, correlation analysis showed that CXCL8 and CXCL1 were positively correlated with neutrophils; CXCL9 and CXCL10 were positively correlated with M1 macrophages. CXCL8 and CXCL1 led to the accumulation of neutrophils in the psoriatic epidermis and dermal infiltration (Armstrong and Read, 2020); the neutrophils gathered at the skin lesions can release IL-17A through the formation of NETs (Lin et al., 2011; Keijsers et al., 2014). In addition, the IL-17A antagonist (secukinumab) used to treat moderate to severe plaque psoriasis can significantly inhibit the secretion of CXCL1 and CXCL8 by keratinocytes, and almost completely eliminate the infiltration of neutrophils in skin lesions (Reich et al., 2015). Previous studies have found the activation and aggregation of macrophages by immunostaining psoriatic lesions, and once macrophages are activated, they can induce psoriasis-like lesions independently of CD4^+^ T cells (Wang et al., 2009). As a key natural immune cell, macrophages are likely to play an important role in the hyperproliferation of keratinocytes (Sa et al., 2007), abnormal angiogenesis (Liu et al., 2015), and inflammation (Morimura et al., 2016) through secretion of IL-20, macrophage migration inhibitory factor (MIF), TNF-α and monocyte chemoattractant protein-1 (MCP-1). In addition, our results reveal the details of the infiltration of 22 immune cells in skin lesions—T cells follicular helper, activated NK cells, and M0 macrophages had a significant positive correlation. The specific mechanism of these correlations requires further experimental evidence.

In order to further explore the relationship between hub genes and the mainstream pathogenic mechanism of psoriasis, we analyzed the relationship between these genes and LCN2/IL17A. It is currently believed that IL17A is the core driving factor of the pathogenesis of psoriasis, and LCN2 plays an indispensable role in this disease by acting as an antibacterial peptide and as a trigger for neutrophil activation in psoriasis (Shao et al., 2016; Frieder et al., 2018). Our analysis showed that the expression level of LCN2/IL17A was significantly positively correlated with the abundance of neutrophils, and the expression of four hub genes (CXCL1, CXCL8, CCL20, and IRF7) was strongly positively correlated with the expression of LCN2/IL17A. This is an interesting discovery. In addition, as mentioned earlier, CXCL1/CXCL8 expression is also strongly correlated with neutrophils. Previous studies have found that LCN2 can activate neutrophils to release pro-inflammatory mediators (such as CXCL8, CXCL10; Shao et al., 2016). As a strong chemokine of neutrophils, CXCL8 binds to its specific receptors CXCR1 and CXCR2, and further mediates the infiltration of neutrophils into the skin lesions. After injection of LCN2 into imiquimod (IMQ)-induced psoriasis-like skin, the mRNA expression levels of IL17A, CXCL1, CCL20, S100A7, and other cytokines and chemokines increased, which supports our analysis results. Although LCN2 injection aggravated the psoriasis-like inflammation induced by IMQ, it did not induce a psoriasis-like phenotype by itself, indicating that LCN2 may need to cooperate with certain stimuli, such as co-enhancing psoriasis with Th17 cytokines inflammatory response (Hau et al., 2016). According to the current immune theory, the activation of multiple dendritic cell subgroups initiates the IL23/Th17/IL17 axis to participate in the pathogenesis of psoriasis (Hawkes et al., 2018). However, this theory cannot fully explain the mechanism of psoriasis, and this activation process is still unclear. Our results show that the expression level of IL17A is strongly positively correlated with the neutrophil fraction, which implies that the initial IL17A is most likely derived from neutrophils. Keijsers et al. (2014) found that the neutrophils in the skin lesions express IL17A-related transcription factor (RoRγt) and contain IL17A mRNA, indicating that neutrophils synthesize IL17A. Lin et al. (2011) found that in psoriasis lesions, the expression level of IL17A in neutrophils and mast cells was even higher than that of lymphocytes. These findings are consistent with our results. Then IL17A acts on keratinocytes to express chemokines (such as CCL20, CXCL1, and CXCL8). CCL20 can specifically recruit CCR6^+^Th17 cells to the psoriasis epidermis, and then produce more IL17A to cause a positive feedback loop (Harper et al., 2009). CXCL1/CXCL8 can recruit more neutrophils to the skin lesions and increase inflammation, which is one of the reasons why psoriasis is a refractory disease. IRF7, as a key transcription factor of IFN-α, plays an important role in the antiviral response of psoriasis (Raposo et al., 2015). Sato et al. investigated the relationship between antimicrobial peptide levels and the clinical outcome of infliximab in patients with psoriasis. It was found that the synergy between S100A7 and CXCL8 may be the cause of resistance to infliximab in patients with psoriasis (Sato et al., 2017). The correlation between S100A7 and CXCL8 was also confirmed in our results. S100A7 functions as an antibacterial peptide and has the ability to induce chemotaxis of neutrophils and T cells, which is closely related to the abnormal differentiation of keratinocytes (Ekman et al., 2017).

Some other genes, although relatively little research in psoriasis, seem to play an important role. CXCR4 is expressed by basal keratinocytes, but its role in psoriasis is still poorly understood. Takekoshi et al. (2013) found and confirmed that up-regulation of CXCR4 can promote the proliferation of keratinocytes in an IL23-mediated psoriatic dermatitis model, which indicates that the up-regulation of CXCR4 expression in skin lesions may contribute to the occurrence of psoriasis. Previous studies have found that the early key event of TNF inhibitors in the remission of psoriasis is the inhibition of the CCR7/CCL19 axis, which strongly supports the key role of the CCR7/CCL19 axis in the pathogenesis of psoriasis (Bosè et al., 2013). Hald et al. (2013) found that the mRNA and protein expression of STAT1 in psoriasis lesions increased, and the levels of the two phosphorylated forms of STAT1 (Tyr701, Ser727) also increased. These data indicate that STAT1 not only changes at the transcription level of psoriasis skin, but also changes at the post-translational level, strongly suggesting that STAT1 plays an important role in the pathogenesis of psoriasis. Recent studies have found that in a mouse model of psoriasis induced by IMQ or IL23, MMP9 inhibitors can significantly reduce skin vasodilation, vascular permeability and symptoms of psoriasis (Chen et al., 2020). These evidences indicate that MMP9 secreted by neutrophils induce skin vasodilation and high permeability by activating skin vascular endothelial cells, promoting the development of psoriatic lesions. This emphasizes the important role of neutrophils and MMP9 in psoriasis. Finally, two envelope proteins (IVL, SPRR1B) are rarely studied in psoriasis, which is novel. What we can know at present is that they are essential in the keratinization and terminal differentiation of keratinocytes (Scholz et al., 2016; Mir et al., 2017). In summary, these genes have an intricate relationship with the pathogenesis of psoriasis, which is worthy of further investigation.

The highlight of this study is to explore the differences in psoriasis gene expression and immune cell infiltration. These hub genes and immune infiltration profiles may be the key to the pathogenesis of psoriasis. Although the expression levels of these hub genes have also been validated in another independent dataset, this study has some limitations. In our future research, if ethical approval is granted, these identified target genes will be verified by RT-qPCR in clinical samples. In addition, the functions of these genes and immune cells need to be further explored through *in vitro* and *in vivo* experiments.

## Conclusion

In summary, the 190 DEGs and 13 hub genes that we identified through bioinformatics analysis are related to the pathogenesis of psoriasis. The biological functions and pathways of the identified genes provide a more detailed molecular mechanism for understanding the occurrence and development of psoriasis. By combining a reliable deconvolution algorithm with large-scale genomic data, we found significant differences in immune infiltration between psoriatic lesions and normal controls. These hub genes and immune cells may provide new insights into the targeted treatment of psoriasis.

## Data Availability Statement

The data analyzed in this article comes from Gene Expression Omnibus (GEO) database (http://www.ncbi.nlm.nih.gov/geo). The accession number can be found in the Materials and Methods section of the article.

## Author Contributions

JJ developed a major research plan. WS and YW analyzed data, draw charts, and wrote manuscripts. BH helped to collected the data and references. The final manuscript read and approved by all authors. All authors contributed to the article and approved the submitted version.

## Conflict of Interest

The authors declare that the research was conducted in the absence of any commercial or financial relationships that could be construed as a potential conflict of interest.

## Footnotes

1 http://www.ncbi.nlm.nih.gov/geo

2 http://kobas.cbi.pku.edu.cn

3 https://metascape.org

4 http://www.genemania.org/

## Supplementary Material

Supplementary Table 1 The top 10 enriched terms of KEGG pathway.

Supplementary Table 2 The top 10 enriched terms via Reaction databases.

## Abbreviations

WHO: The World Health Organization
GEO: Gene Expression Omnibus
DEGs: differentially expressed genes
PPI: protein − protein interaction
LS: lesions
NS: normal skin
NL: non-lesion
FDR: false discovery rate
FC: fold-changes
GSEA: Gene set enrichment analysis
TF: Transcription factor
Tregs: regulatory T cells
MIF: macrophage migration inhibitory factor
MCP-1: monocyte chemoattractant protein-1
IMQ: imiquimod.
