# Supplementary material for: Identification of Hub Genes and Immune Infiltration in Psoriasis by Bioinformatics Method
Source: Front Genet. 2021 Feb 3;12:606065. doi: 10.3389/fgene.2021.606065 (PMC7886814; doi:10.3389/fgene.2021.606065)
Supplement: Supplementary Table 1 — The top 10 enriched terms of KEGG pathway. [file Data_Sheet_1.DOCX]

| Term | P.adjust | Genes | Count |
| --- | --- | --- | --- |
| hsa04062: Chemokine signaling pathway | 8.66E-06 | CXCL1, CCR7, CCL20, CXCR4, CXCL13, CXCL9, CXCL8, CXCR2, STAT1, CCL18, CCL27, CXCL10 | 12 |
| hsa04060: Cytokine-cytokine receptor interaction | 1.03E-04 | CXCL1, CCR7, CCL20, CXCR4, CXCL13, IL19, CXCL9, CXCL8, CXCR2, CCL18, CCL27, CXCL10 | 12 |
| hsa05164: Influenza A | 1.71E-04 | IRF7, PRSS3, CXCL8, RSAD2, OAS1, OAS2, MX1, STAT1, TMPRSS4, CXCL10 | 10 |
| hsa05160: Hepatitis C | 0.00435 | CLDN8, IFIT1, IRF7, CXCL8, OAS1, OAS2, STAT1 | 7 |
| hsa04668: TNF signaling pathway | 0.007913 | CXCL1, NOD2, CCL20, MMP9, SELE, CXCL10 | 6 |
| hsa05219: Bladder cancer | 0.011754 | TYMP, MMP9, CXCL8, MMP1 | 4 |
| hsa05202: Transcriptional misregulation in cancer | 0.012794 | PLAT, NFKBIZ, MMP9, BCL2A1, CXCL8, GZMB, ZBTB16 | 7 |
| hsa04621: NOD-like receptor signaling pathway | 0.027007 | CXCL1, NOD2, CXCL8, CARD6 | 4 |
| hsa00240: Pyrimidine metabolism | 0.029608 | TYMP, RRM2, UPP1, PNP, CMPK2 | 5 |
| hsa04620: Toll-like receptor signaling pathway | 0.034504 | IRF7, CXCL9, CXCL8, STAT1, CXCL10 | 5 |
