# Supplementary material for: Identification of Hub Genes and Immune Infiltration in Psoriasis by Bioinformatics Method
Source: Front Genet. 2021 Feb 3;12:606065. doi: 10.3389/fgene.2021.606065 (PMC7886814; doi:10.3389/fgene.2021.606065)
Supplement: Supplementary Table 2 — The top 10 enriched terms via Reaction databases. [file Data_Sheet_2.DOCX]

| Term | P.adjust | Genes | Count |
| --- | --- | --- | --- |
| R-HSA-168256: Immune System | 1.23E-24 | LCN2, IFI27, MX1, PRSS3, S100A12, IFIT3, RSAD2, IFIT1, CD207, FLG2, ZBTB16, STAT1, S100A9, OAS1, CXCR2, OAS2, ISG15, S100A8, NOD2, IL36A, CCL20, S100A7, CST6, GGH, IL36G, HPSE, IL36RN, SERPINB3, OASL, IL37, LTF, CXCL10, LRG1, PI3, CXCL8, CXCL1, IL19, PNP, IRF7, IFI6, CD274, GM2A, TCN1, CDC20, S100A7A, BTC, MMP9, KIF20A, MMP1 | 49 |
| R-HSA-6809371: Formation of the cornified envelope | 3.15E-19 | TGM1, KRT77, IVL, LCE3D, KLK13, SPRR1A, KRT79, SPRR3, DSC2, KRT16, KRT6B, KRT6A, DSG3, SPRR1B, PI3, SPRR2G, SPRR2B | 17 |
| R-HSA-1280215: Cytokine Signaling in Immune system | 2.42E-16 | LCN2, IFI27, MX1, S100A12, IFIT3, RSAD2, IFIT1, STAT1, OAS1, OAS2, ISG15, NOD2, IL36A, CCL20, IL36G, IL36RN, OASL, IL37, CXCL10, CXCL1, IL19, IRF7, IFI6, CXCL8, BTC, MMP9, MMP1 | 27 |
| R-HSA-6805567: Keratinization | 4.03E-16 | TGM1, KRT77, IVL, LCE3D, KLK13, SPRR1A, KRT79, SPRR3, DSC2, KRT16, KRT6B, KRT6A, DSG3, SPRR1B, PI3, SPRR2G, SPRR2B | 17 |
| R-HSA-909733: Interferon alpha/beta signaling | 2.10E-13 | IRF7, IFI6, IFI27, OASL, MX1, OAS1, OAS2, ISG15, IFIT3, RSAD2, IFIT1 | 11 |
| R-HSA-380108: Chemokine receptors bind chemokines | 3.96E-13 | CXCL1, CXCR4, CXCL9, CXCL8, CCR7, CXCR2, CXCL10, CCL20, CXCL13, CCL27 | 10 |
| R-HSA-6798695: Neutrophil degranulation | 4.48E-13 | CXCL1, HPSE, S100A7, SERPINB3, LCN2, PNP, GM2A, TCN1, FLG2, CXCR2, PRSS3, S100A9, LTF, MMP9, LRG1, S100A12, CST6, GGH, S100A8 | 19 |
| R-HSA-168249: Innate Immune System | 1.08E-11 | LCN2, PRSS3, S100A12, LRG1, FLG2, CXCR2, ISG15, S100A8, NOD2, S100A9, S100A7, CST6, GGH, HPSE, LTF, SERPINB3, PI3, CXCL1, IRF7, PNP, GM2A, TCN1, S100A7A, MMP9 | 24 |
| R-HSA-6799990: Metal sequestration by antimicrobial proteins | 5.18E-11 | LCN2, S100A7A, S100A9, LTF, S100A7, S100A8 | 6 |
| R-HSA-913531: Interferon Signaling | 2.57E-10 | IRF7, STAT1, IFI6, IFI27, OASL, MX1, OAS1, OAS2, ISG15, IFIT3, RSAD2, IFIT1 | 12 |
